# Supplementary material for: Effects of individualized brain anatomies and EEG electrode positions on inferred activity of the primary auditory cortex
Source: Front Neuroinform. 2022 Oct 13;16:970372. doi: 10.3389/fninf.2022.970372 (PMC9606706; doi:10.3389/fninf.2022.970372)
Supplement: Supplementary file 1 [file Image_1.pdf]

Supplementary Material

1 SUPPLEMENTARY FIGURE

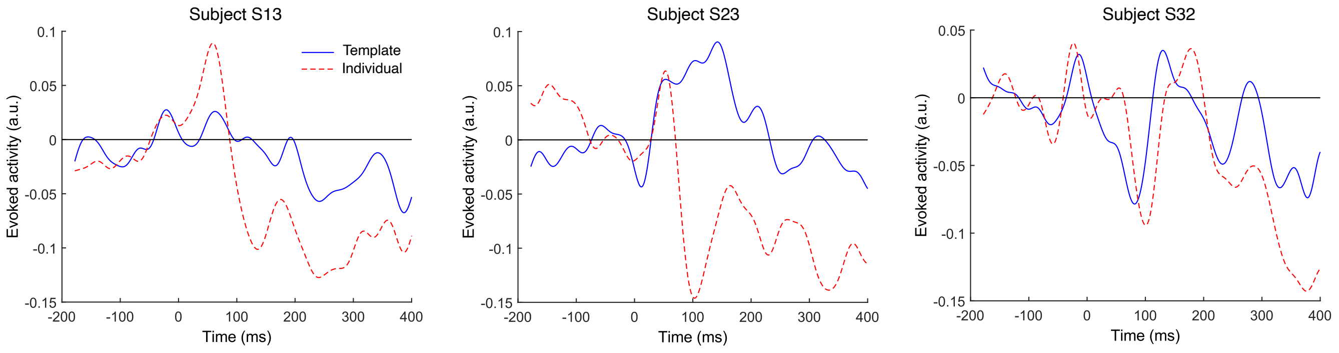

Figure S1. Evoked PAC activity of the left hemisphere inferred via dSPM for the three removed subjects. No distinct P1 or N1 peaks are found using template anatomies and electrode locations (blue). Using a fully individualized configuration (red), however, does yield very prominent peaks.
